# Human and Murine Innate Immune Cell Populations Display Common and Distinct Response Patterns during Their *In Vitro* Interaction with the Pathogenic Mold *Aspergillus fumigatus*

**DOI:** 10.3389/fimmu.2017.01716

**Published:** 2017-12-06

**Authors:** Anna-Maria Hellmann, Jasmin Lother, Sebastian Wurster, Manfred B. Lutz, Anna Lena Schmitt, Charles Oliver Morton, Matthias Eyrich, Kristin Czakai, Hermann Einsele, Juergen Loeffler

**Affiliations:** ^1^Medizinische Klinik & Poliklinik II, Universitätsklinikum Würzburg, Würzburg, Germany; ^2^Institute of Virology and Immunobiology, University of Würzburg, Würzburg, Germany; ^3^School of Science and Health, Western Sydney University, Campbelltown, NSW, Australia; ^4^Kinderklinik und Poliklinik, Universitätsklinikum Würzburg, Würzburg, Germany

**Keywords:** murine model, humans, *Aspergillus fumigatus*, innate immune response, fungal infection

## Abstract

*Aspergillus fumigatus* is the main cause of invasive fungal infections occurring almost exclusively in immunocompromised patients. An improved understanding of the initial innate immune response is key to the development of better diagnostic tools and new treatment options. Mice are commonly used to study immune defense mechanisms during the infection of the mammalian host with *A. fumigatus*. However, little is known about functional differences between the human and murine immune response against this fungal pathogen. Thus, we performed a comparative functional analysis of human and murine dendritic cells (DCs), macrophages, and polymorphonuclear cells (PMNs) using standardized and reproducible working conditions, laboratory protocols, and readout assays. *A. fumigatus* did not provoke identical responses in murine and human immune cells but rather initiated relatively specific responses. While human DCs showed a significantly stronger upregulation of their maturation markers and major histocompatibility complex molecules and phagocytosed *A. fumigatus* more efficiently compared to their murine counterparts, murine PMNs and macrophages exhibited a significantly stronger release of reactive oxygen species after exposure to *A. fumigatus*. For all studied cell types, human and murine samples differed in their cytokine response to conidia or germ tubes of *A. fumigatus*. Furthermore, Dectin-1 showed inverse expression patterns on human and murine DCs after fungal stimulation. These specific differences should be carefully considered and highlight potential limitations in the transferability of murine host–pathogen interaction studies.

## Introduction

Humans inhale hundreds of airborne fungal spores daily, including spores from the saprophytic mold *Aspergillus fumigatus*, which is ubiquitous in the environment. While the fungus rarely causes diseases in healthy individuals, disorders of the immune system are associated with a wide spectrum of *Aspergillus*-related diseases (1). Overshooting immune response to the fungus can lead to hypersensitivity syndromes such as allergic bronchopulmonary aspergillosis, whereas invasive aspergillosis (IA) is a major cause of morbidity and mortality in immunocompromised patients.

The mammalian immune system, evolving under continuous exposure to airborne fungal spores, possesses a vast arsenal of strategies to combat invading fungi including mediating tolerance to commensals and limiting hyperinflammation to prevent tissue damage. In the absence of an effective immune response, *Aspergillus* conidia swell and become invasive by germinating into the lung tissue and entering the blood stream. Alveolar macrophages act as the first line of defense in the airways by phagocytosis of conidia and secretion of pro-inflammatory cytokines and reactive oxygen species (ROS) (2). Neutrophil granulocytes [polymorphonuclear cells (PMNs)] also play a major role in the early immune defense against IA, as they are able to prevent germination and kill fungal hyphae through the release of ROS, phagocytosis, or formation of neutrophil extracellular traps. Dendritic cells (DCs) represent an important bridge between innate and adaptive immunity as they process fungal antigens and subsequently stimulate specific T-cells *via* antigen-presentation by major histocompatibility complex (MHC) I and II molecules. They also orchestrate the immune response by secreting an array of pro- and anti-inflammatory cytokines. Stimulation of pattern recognition receptors (PRRs), such as toll-like receptors (TLR)-2 and -4 (3) and the Dectin-1-receptor, is crucial to the activation of these immune cell subsets (4).

Detailed insights into the pathophysiology of the infection and mechanisms of the host–pathogen interaction are urgently needed to facilitate development of new prophylactic and therapeutic tools and strategies. Due to their easy accessibility, relatively short generation time, and availability of genetically defined strains, mouse models are commonly used to characterize the interaction of *A. fumigatus* with the mammalian host *in vivo* and to evaluate novel therapeutic strategies. There is, however, increasing evidence of major functional differences between the human and murine immune systems, indicating limited application of data obtained in studies employing murine cells or models (5, 6). On one hand, the composition of the murine leukocyte repertoire differs from that of humans; in mice only 7–28% of peripheral blood leukocytes are PMNs in contrast to 35–70% in human blood (7). On the other hand, functional differences, such as the sequence of participating immunological cells after antigen challenge as well as the dose of antigen needed to initiate immunological reaction in delayed type hypersensitivity have been described (5).

So far, little is known about functional differences in the human and murine immune defense against *A. fumigatus*. Thus, this study sought to provide a comparative functional assessment between human and murine innate immune cell subsets routinely employed in host–pathogen interaction studies (8–13). Our data demonstrate that mice and men which differ in their size, habitat, lifespan, genome size, and blood composition possess principle differences in how selected innate immune cell populations specifically interact with the pathogenic mold *A. fumigatus*.

## Materials and Methods

### Fungal Strains and Cultivation Conditions

*Aspergillus fumigatus* strain American Type Culture Collection 46645 was incubated on beer wort agar (Institute of Hygiene and Microbiology, University of Wuerzburg, Germany) for 72 h at 37°C. Conidial suspensions were prepared by rinsing plates with sterile water and filtered through a 40-µm cell strainer (BD Falcon™ Cell Strainer, BD Biosciences). To generate germ tubes, 1 × 10^8^ resting conidia were inoculated in Rosewell Park Memorial Institute (RPMI) 1640 Medium (Life technologies) and cultured at 37°C until the germ tubes reached a length of about 10–30 µm. To preserve fungal morphotypes during coculture experiments, conidia and germ tubes were inactivated with 100% ethanol (Sigma) for 30 min, washed five times with sterile water, and stored in RPMI at −20°C. For time-lapse microscopy red fluorescent *A. fumigatus* conidia and germ tubes were generated as described before (9).

### Isolation and Culture of Human and Murine Immune Cells

#### Dendritic Cells

Peripheral blood mononuclear cells were isolated from leukocyte concentrates from healthy human donors (Institute of Transfusion Medicine, University Hospital of Wuerzburg) by Ficoll (Biochrome) density centrifugation. After positive magnetic selection of CD14^+^ cells (CD14 MicroBeads, Miltenyi Biotec), monocytes were cultured for 5 days in RPMI 1640 medium supplemented with 10% fetal calf serum (FCS) (Sigma), rGM-CSF (100 ng/ml, Bayer), and rIL4 (10 ng/ml, Miltenyi Biotec) (9).

Murine bone marrow derived dendritic cells (BMDCs) were generated as described before (14). Briefly, bone marrow from healthy mice was harvested and cultured in R10-medium (RPMI 1640) (GIBCO BRL) with 100 U/ml Penicillin (Sigma), 100 µg/ml Streptomycin (Sigma), 2 mM l-glutamine (Sigma), 50 µM 2-mercaptoethanol (Sigma), 10% FCS (PAA), and 200 U/ml rmGM-CSF (Reprotech/Tebu) for 6 days. The non-adherent cells were harvested and cultured in R10-medium without cytokines.

#### Polymorphonuclear Cells

To isolate human PMNs anticoagulated blood from healthy human donors was layered on a polysaccharide gradient (Polymorphprep, Axis Shield). After 30 min of centrifugation (500*g*), the PMN interphase was harvested and pelleted for 5 min at 300*g*. Remaining erythrocytes were lysed with EL buffer (Qiagen). Isolation of murine PMNs, which were obtained from bone marrow of C57BL/6NCrl mice, was performed using the EasySep™ Mouse Neutrophil Enrichment Kit (Stem Cell Technologies) according to the manufacturer’s protocol. Both murine and human PMNs were cultured in RPMI 1640 Medium with 5% FCS.

#### Macrophages

Human monocytes obtained from healthy subjects as described above were cultured in RPMI 1640 medium supplemented with 10% FCS and rM-CSF (Immunotools) for 6 days. Only adherent cells were used for experiments. To generate murine macrophages, bone marrow cells of healthy mice were cultured in R10-medium with M-CSF supernatant [mouse M-CSF (Immunotools) supernatant, L929] for 6 days. Adherent cells were harvested and suspended in R10-medium.

### Time-Lapse Video Microscopy

3.5 × 10^4^ human monocyte-derived immature dendritic cells (moDCs) or murine BMDCs were stimulated with 3.5 × 10^5^ *A. fumigatus* (Afu-dTomato) conidia or germ tubes [multiplicity of infection (MOI) = 10]. Polystyrene beads (Sigma) were used as an unspecific stimulus. Image acquisition over a time period of 3 h was performed using a Leica AF6000 time-lapse microscope with a picture frequency of 5/min. Image analysis was conducted with LAS AF lite (Leica), ImageJ1.45s (Wayne Rasband), and Irfan View 4.32 (Irfan Skiljan) software. For each stimulus, phagocytosis activity was analyzed in six independent movies, respectively, following moDCs or BMDCs over a time period of 3 h by separately counting phagocytosed and extracellular particles or fungi, respectively.

### Flow Cytometry

Dendritic cells were stimulated with inactivated *A. fumigatus* conidia and germ tubes (MOI = 1), 100 µg/ml zymosan depleted [a yeast cell wall preparation, which was treated with hot alkali to remove all TLR-stimulating properties to selectively activate Dectin-1 (dZym, InvivoGen)] or 1 mg/ml lipopolysaccharid (LPS, Sigma) for 24 h. Subsequently, cells were harvested, washed, and resuspended in cold Hank’s balanced salt solution (HBSS, Sigma) containing 2 mM EDTA (Sigma). The following antibodies were used for extracellular staining: HLA-ABC-PE (BD Biosciences), HLA-DR-PE (BD Biosciences), CD1a-APC (BD Biosciences), CD14-FITC (BD Biosciences), CD80-APC (Miltenyi Biotec), CD86-PE (BD Biosciences), Dectin-1-PE (R&D) (human cells) and HLA-2K^b^-FITC (BD Biosciences), HLA-Ia/I-E-PE (BioLegend), CD11c-APC (BioLegend), CD80-FITC (BioLegend), CD86-PE (BD Biosciences), and Dectin-1-PE (R&D) [murine cells]. 5 × 10^5^ cells were stained for 15 min at 4°C. Subsequently, cells were washed twice and 10^4^ viable cells according to FSC-SSC properties were acquired using a FACS Calibur (BD Biosciences) flow cytometer and CellQuest Pro software (version 5.2). Data were analyzed with FlowJo software (Tree Star Inc., Ashland, OR, USA).

### ROS Quantification

Polymorphonuclear cells and macrophages were stimulated with inactivated *A. fumigatus* conidia and germ tubes (MOI = 1), 100 µg/ml zymosan depleted or 10 µg/ml phorbol-myristate-acetate [the major endogenous ROS inducer in PMNs and macrophages (PMA, Sigma-Aldrich)]. ROS-formation in human and murine PMNs and macrophages was determined by ROS-dependent CM-H_2_DCF oxidation (dichlorfluorescein, Sigma-Aldrich). Excitation was performed at 485 nm, and fluorescence emission was detected at 535 nm (GENios microplate reader, TECAN). PMNs were stimulated at 37°C for 1 h and macrophages for 2.5 h before measuring fluorescence intensity.

### Multiplex Cytokine Assays

Polymorphonuclear cells (2 × 10^6^/ml), macrophages (8 × 10^5^/ml), and DCs (1 × 10^6^/ml) were stimulated with *A. fumigatus* conidia and germ tubes (MOI = 1), zymosan depleted (100 µg/ml), LPS (1 µg/ml), or plain culture medium (negative control). Supernatants were harvested after 3 h (PMNs), 12 h (macrophages), or 24 h (DCs) and stored at −20°C. Cytokine concentrations (IL1β, IL4, IL6, huIL8, muGroα, IL10, IL12p70, IL18, IL23, IP10, MCP1, MIP1α, MIP1β, TNFα) were quantified with a human and mouse 13-Plex panel assay (Affymetrix, eBioscience).

### Fungicidal Activity of PMNs

Polymorphonuclear cell were isolated from whole blood of four healthy human donors and two mice as described above. PMNs were diluted in colorless RPMI + 10% FCS at a concentration of 2 × 10^6^/ml. 5 × 10^5^ PMNs were seeded per well of a 24-well plate and 5 × 10^5^ vital *A. fumigatus* germ tubes were added (MOI = 1, ideal MOI was determined in a preceding experiment). Control wells containing only PNMs or fungal cells as well as blank wells containing culture medium without cells were prepared. After 2 and 4 h of coculture at 37°C, hypotonic lysis of PMNs was performed by washing wells twice with 1,000 µl cold distilled water, followed by 5 min incubation on ice. Supernatants were carefully removed, and 200 µl HBSS supplemented with 400 µg/ml of 2,3-bis-(2-methoxy-4-nitro-5-sulphenyl)-(2H)-tetrazolium-5-carboxanilide [XTT (Sigma)] and 50 µg/ml of coenzyme (Sigma) were added. After 90 min incubation at 37°C and centrifugation at 300*g* for 5 min, 100 µl supernatant of each well were transferred to a 96-well plate and OD_450_ was measured in a microplate reader. Fold changes of fungal XTT metabolism were calculated according to the following formula: $\text{Fold change }=({\text{OD}}_{{\;}_{450}}^{{\;}^{\text{PMN}+\mathrm{Fungus}}}-{\text{OD}}_{{\;}_{450}}^{{\;}^{\mathrm{PMN}}})/({\text{OD}}_{{\;}_{450}}^{{\;}^{\mathrm{Fungus}}}-{\text{OD}}_{{\;}_{450}}^{{\;}^{\mathrm{Blank}}}).$

### Statistics

Significance testing was performed using GraphPad Prism 7 (Graphpad Software, Inc.) using different statistical test which are noted in each figure legend. Statistical significance is denoted as follows: *p < 0.05, **p < 0.01, and ***p < 0.001.

## Results

### Murine Neutrophils and Macrophages Show Stronger ROS Release upon Stimulation with *A. fumigatus* Germ Tubes and Depleted Zymosan (dZym)

**Figure 1:**
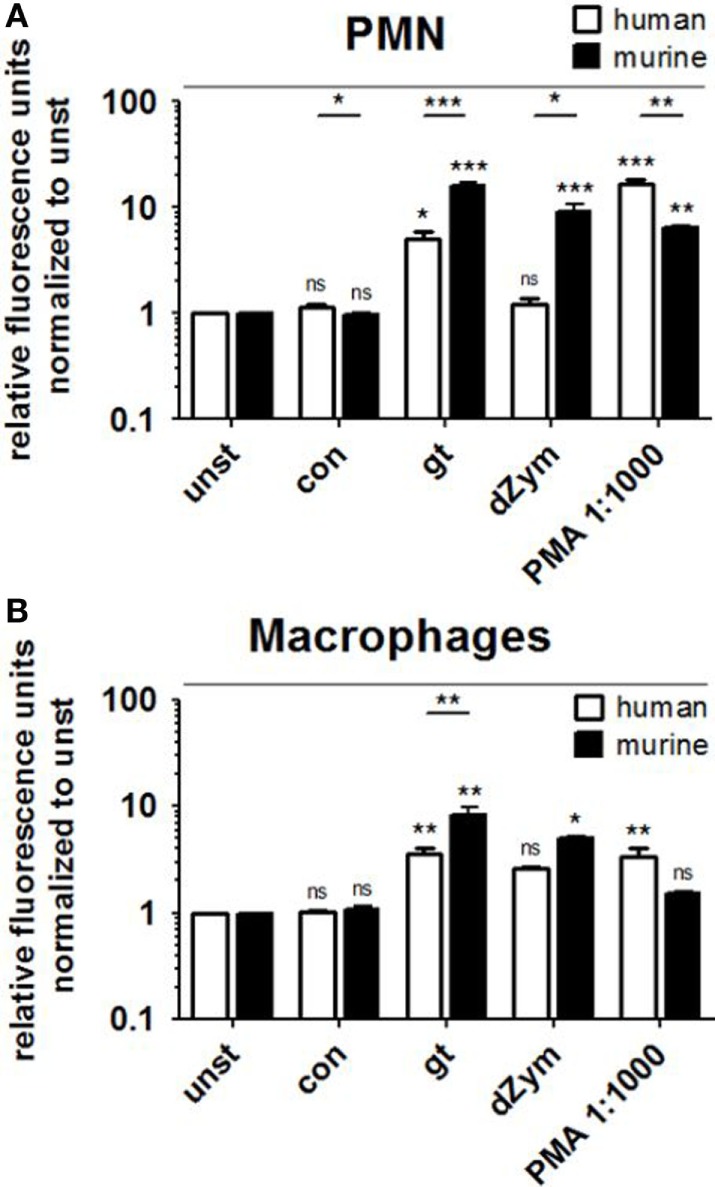
Murine polymorphonuclear cells (PMNs) and macrophages exhibit a stronger reactive oxygen species (ROS) response to *Aspergillus fumigatus* germ tubes and depleted zymosan (dZym). Human and murine PMNs **(A)** or macrophages **(B)** were stimulated with *A. fumigatus* conidia (con), germ tubes (gt), dZym, or phorbol 12-myristate 13-acetate (PMA). ROS production was measured by a dichlorfluorescein-based assay. Mean fold changes of relative fluorescence units after 2 (PMNs) and 3 h (macrophages) are shown. Seven human and three murine samples were analyzed. Significance was calculated as difference to unstimulated with ANOVA and Tukey post-test (stars above of bars) as well as between human and mouse specimens with unpaired *t*-Test, Error bars indicate SDs.

Reactive oxygen species production by human and murine neutrophils and macrophages cocultured with *A. fumigatus* conidia or germ tubes was quantified and compared. No significant induction of oxidative burst in PMNs or macrophages of either species was observed upon conidial stimulation. *A. fumigatus* germ tubes and dZym (binding exclusively to dectin-1) led to significantly higher ROS release by murine PMNs (Figure 1A) and macrophages (Figure 1B). By contrast, human PMNs and macrophages released significantly more ROS after stimulation with the major endogenous ROS inducer PMA.

### Murine PMNs Exert Stronger and Broader Pro-inflammatory Cytokine Response to *A. fumigatus* Germ Tubes and dZym

**Figure 2:**
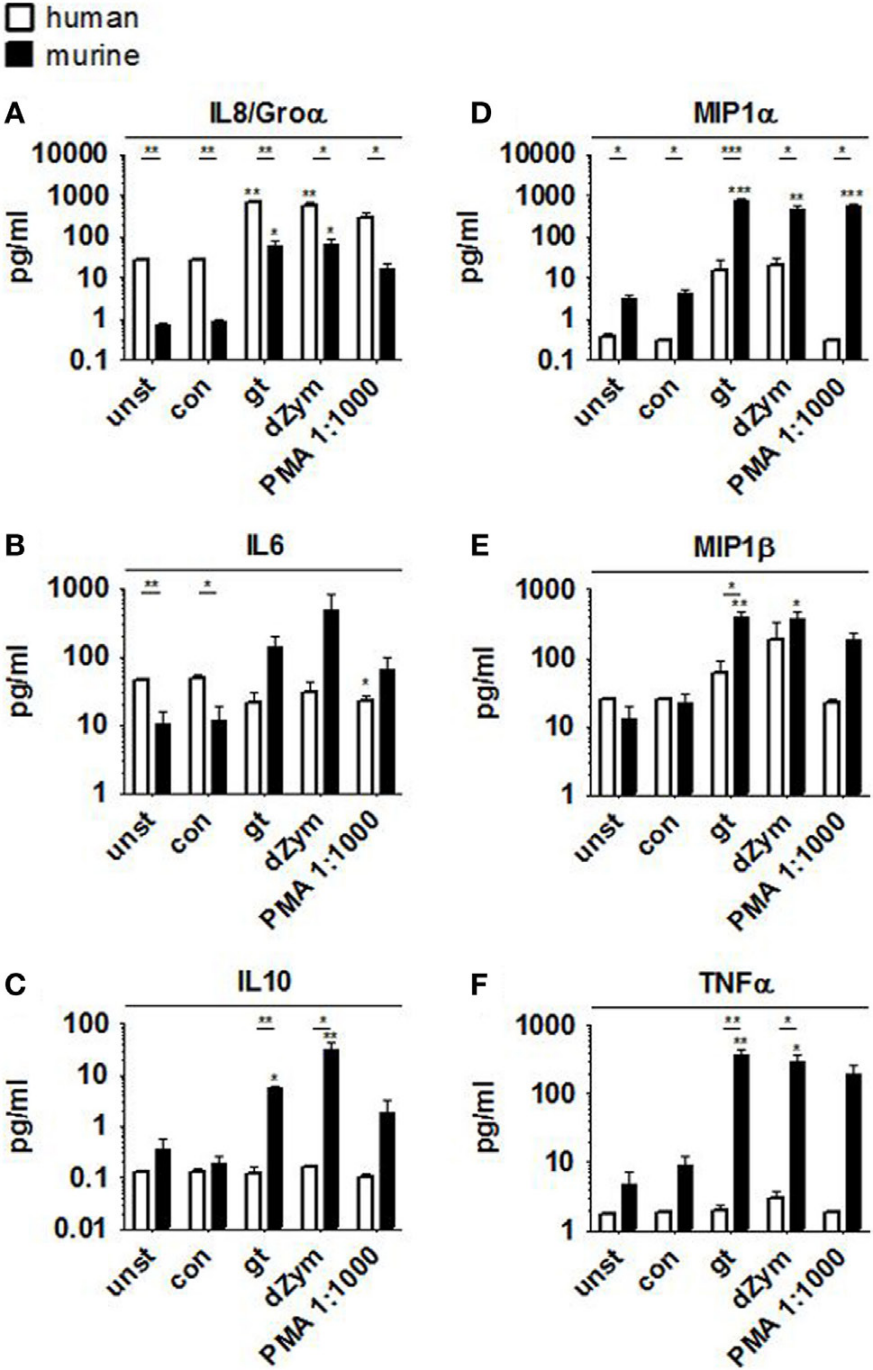
*Aspergillus fumigatus* germ tubes and depleted zymosan (dZym) trigger strong pro-inflammatory cytokine release by murine neutrophils. Murine or human neutrophils were stimulated with *A. fumigatus* conidia, germ tubes, dZym or PMA for a period of 3 h. Subsequently, cytokine concentrations in the culture medium were determined by 13-plex cytokine assays. PMNs obtained from three human donors (white bars) and three mice (black bars) were assessed. Mean concentrations of IL8/Groα **(A)**, IL6 **(B)**, IL10 **(C)**, MIP1α **(D)**, MIP1β **(E)**, and TNFα **(F)** are shown. Error bars indicate standard deviations. Significance was calculated as difference to unstimulated with ANOVA and Tukey post-test (stars above of bars) as well as between human and mouse specimens with unpaired *t*-Test.

Strong ROS response was paralleled by the secretion of pro-inflammatory cytokines Groα, IL6, MIP1α, MIP1β, and TNFα by murine PMNs after stimulation with *A. fumigatus* germ tubes or dZym, as well as the anti-inflammatory cytokine IL10 (Figure 2). Pro-inflammatory cytokine release was also significantly induced by PMA stimulation, whereas coculture with *A. fumigatus* conidia did not result in significant induction of MIP1β or TNFα secretion. Human PMNs showed strongly elevated IL8 release when stimulated with *A. fumigatus* germ tubes, dZym or PMA, while MIP1α and MIP1β secretion was weakly induced by *A. fumigatus* germ tubes and dZym.

### Human and Murine Macrophages Release Distinct Cytokine Patterns When Cocultured with *A. fumigatus* or Stimulated with Synthetic Agonists

**Figure 3:**
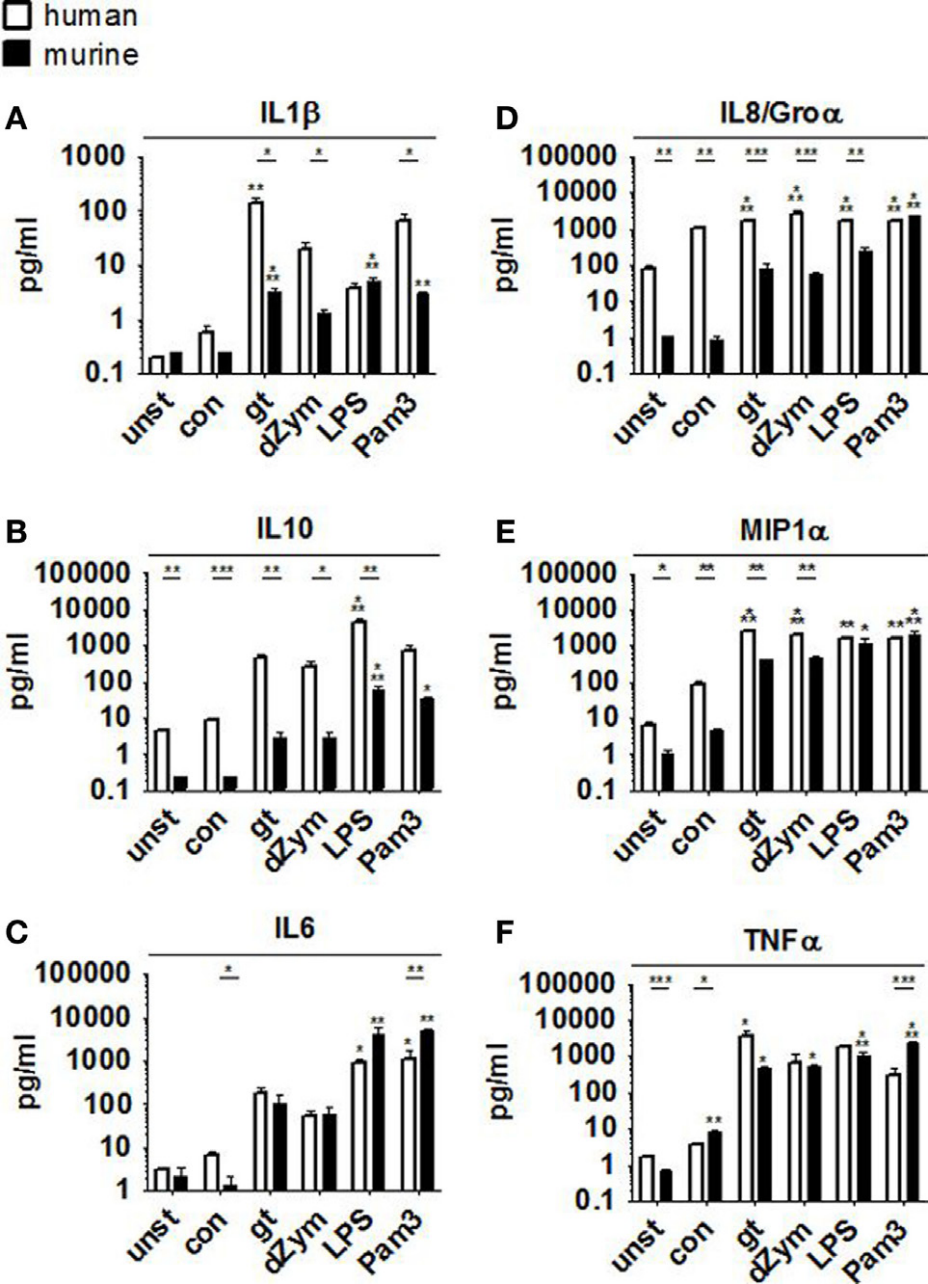
Distinct cytokine response pattern to *Aspergillus fumigatus* by human and murine macrophages. Cytokine patterns in culture supernatants of human and murine macrophages were studied by a 13-plex cytokine assay upon 12 h stimulation with *A. fumigatus* conidia or germ tubes as well as depleted zymosan (dZym), LPS, or Pam3CSK. Four human (white bars) and four murine (black bars) samples were assessed. Mean concentrations of IL1β **(A)**, IL10 **(B)**, IL6 **(C)**, IL8/Groα **(D)**, MIP1α **(E)**, and TNFα **(F)** are shown. Error bars indicate standard deviations. Significance was calculated as difference to unstimulated with ANOVA and Tukey post-test (stars above of bars) as well as between human and mouse specimens with unpaired *t*-test.

Pro- and anti-inflammatory cytokine secretion was strongly induced in both murine and human macrophages upon coculture with *A. fumigatus* germ tubes, dZym, or synthetic agonists LPS and Pam3CSK. While human macrophages released higher concentrations of MIP1α, IL8, and IL10 (Figures 3B,D,E), fold changes of IL8 and MIP1α were higher in murine samples due to differences in baseline levels. In comparison to murine cells stimulation of human macrophages by *A. fumigatus* germ tubes, dZym, and Pam3CSK resulted in significantly greater IL1β release (Figure 3A). Interestingly, a small but significant increase in MIP1α and TNFα (Figures 3E,F) secretion by macrophages of both species was observed upon conidial stimulation. In human macrophages, this was paralleled by an even more pronounced upregulation of IL8 release and minor elevations of IL1β and IL6 (Figures 3A,C,D).

### Human moDCs Show Higher Phagocytosis Rates of *A. fumigatus* Conidia and Germ Tubes

**Figure 4:**
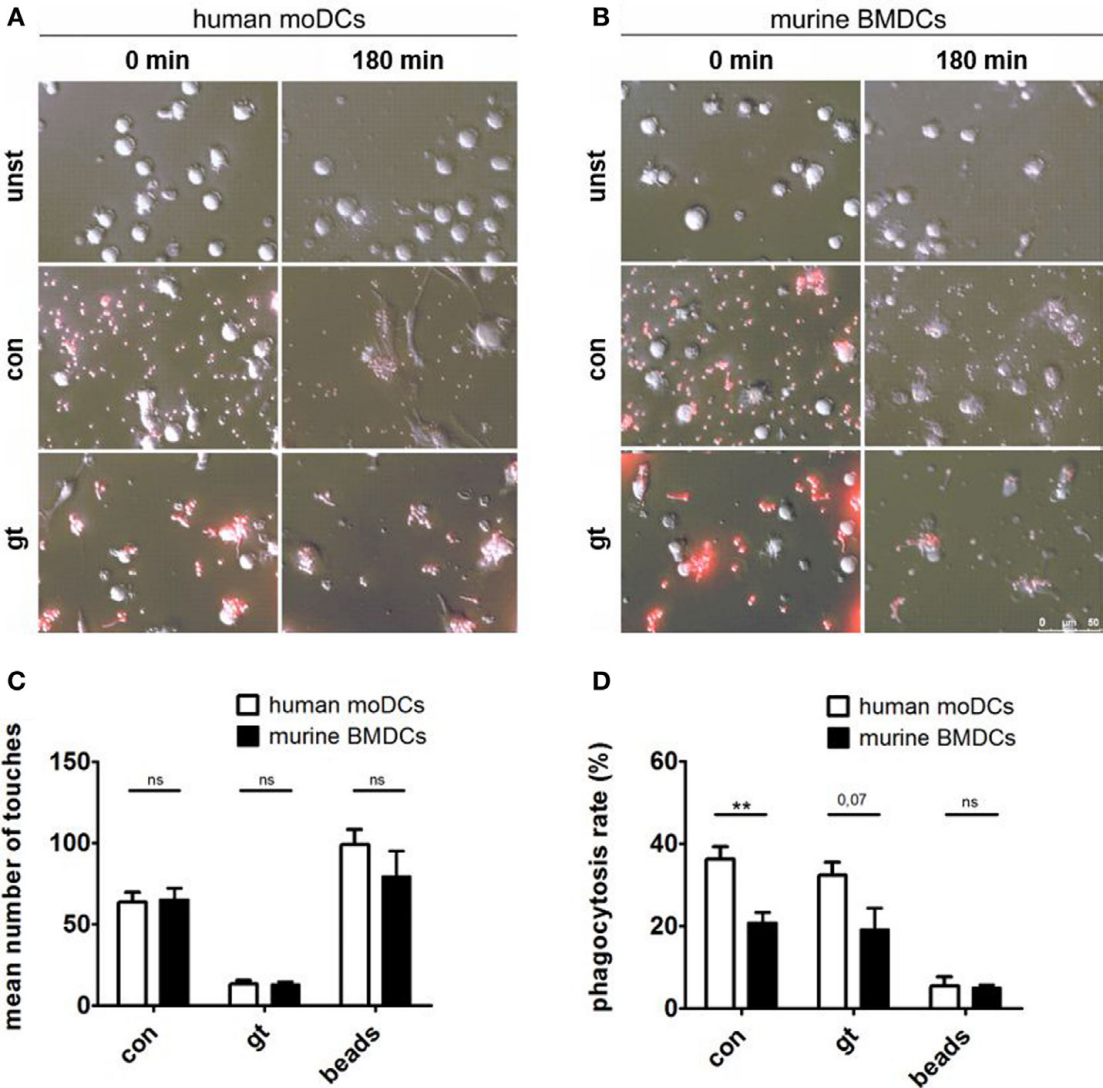
Human dendritic cells (DCs) contact and phagocytose *Aspergillus fumigatus* conidia and germ tubes more effectively. Representative images show phagocytosis of red fluorescent conidia and germ tubes by human **(A)** and murine **(B)** DCs at the indicated time points (duration of coculture). Number of touches **(C)** of human and murine DCs with *A. fumigatus* conidia (con), germ tubes (gt), and polystyrene beads (beads) and phagocytosis rates **(D)** were determined by live imaging analysis. Mean values of touched **(C)** or mean percentage of phagocytosed **(D)** fungal cells or beads after 3 h of coculture are given in the figure. Six human and murine donors were assessed. Significance was calculated with unpaired *t*-Test and error bars indicate SDs.

Live imaging was performed to visualize the interaction between human (Figure 4A) or murine DCs (Figure 4B) with *A. fumigatus* conidia or germ tubes. Analyzing the number of touches of DCs and fungal cells or the unspecific polystyrene bead control revealed no significant differences between human and murine cells over 3 h (Figure 4C). Assessing the percentage of phagocytosed *A. fumigatus* conidia or germ tubes after 3 h, however, human moDCs showed higher phagocytosis rates than murine BMDCs, whereas the internalization of polystyrene beads by human and murine DCs did not differ significantly (Figure 4D).

### Dectin-1 Is Inversely Regulated on Human and Murine DCs after Stimulation with *A. fumigatus* Conidia and Germ Tubes

**Figure 5:**
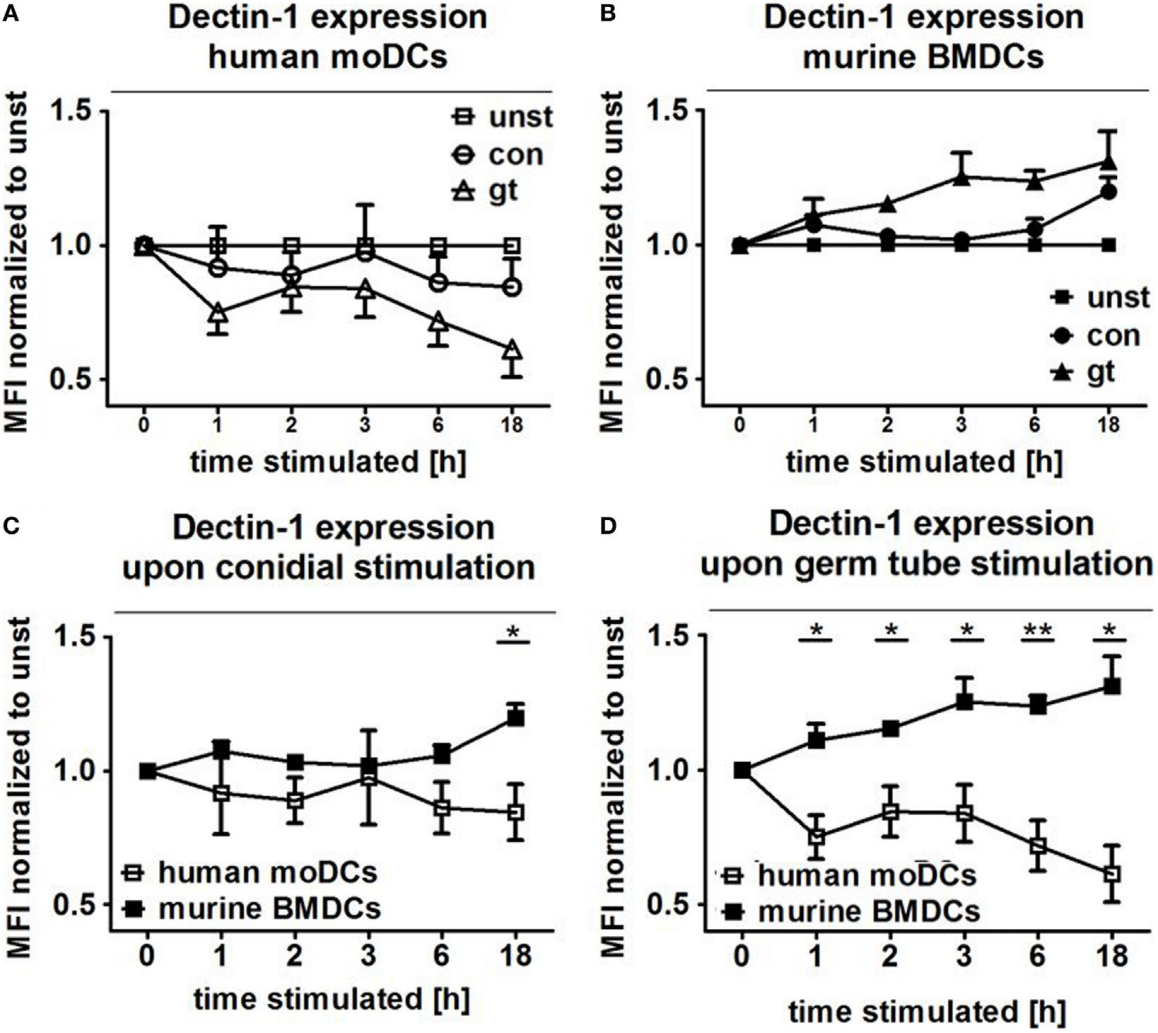
Human and murine dendritic cells show inverse Dectin-1 surface expression patterns after exposure to *Aspergillus fumigatus*. Dectin-1 expression on the cell surface of human moDCs **(A)** and murine bone marrow derived dendritic cells (BMDCs) **(B)** was analyzed by flow cytometry after coculture with *A. fumigatus* conidia and germ tubes for different periods. Mean fold changes (±SDs) of mean fluorescence intensity (MFI) compared to unstimulated cells are given in the figure. Two murine and four human samples were analyzed. The lower panels directly compare Dectin-1 expression following exposure of human moDCs and murine BMDCs to *A. fumigatus* germ tubes **(C)** and conidia **(D)**. Human and murine samples were compared with unpaired *t*-Test, error bars show SDs.

The transmembrane glycoprotein Dectin-1, also known as C-type lectin domain family member 7A, plays an important role as an *A. fumigatus* phagocytosis receptor (4). Thus, Dectin-1 expression on DCs cocultured with ethanol-inactivated conidia and germ tubes for different periods was assessed and compared with unstimulated DCs. After only 1 h of coculture with both fungal morphotypes, surface expression of Dectin-1 started to decline on human moDCs, with a more pronounced effect after stimulation with germ tubes (Figure 5A). By contrast, increased Dectin-1 expression was observed on murine BMDCs stimulated with *A. fumigatus* germ tubes (Figure 5B). For each of the coculture periods studied, a significant difference in Dectin-1 surface expression was detected between human and murine DCs stimulated with *A. fumigatus* germ tubes, whereas prolonged exposure (18 h) to inactivated conidia was necessary to observe significantly different expression (Figures 5C,D).

### Human and Murine DCs Cocultured with *A. fumigatus* Show Distinct Surface Antigen and Cytokine Response Patterns

**Figure 6:**
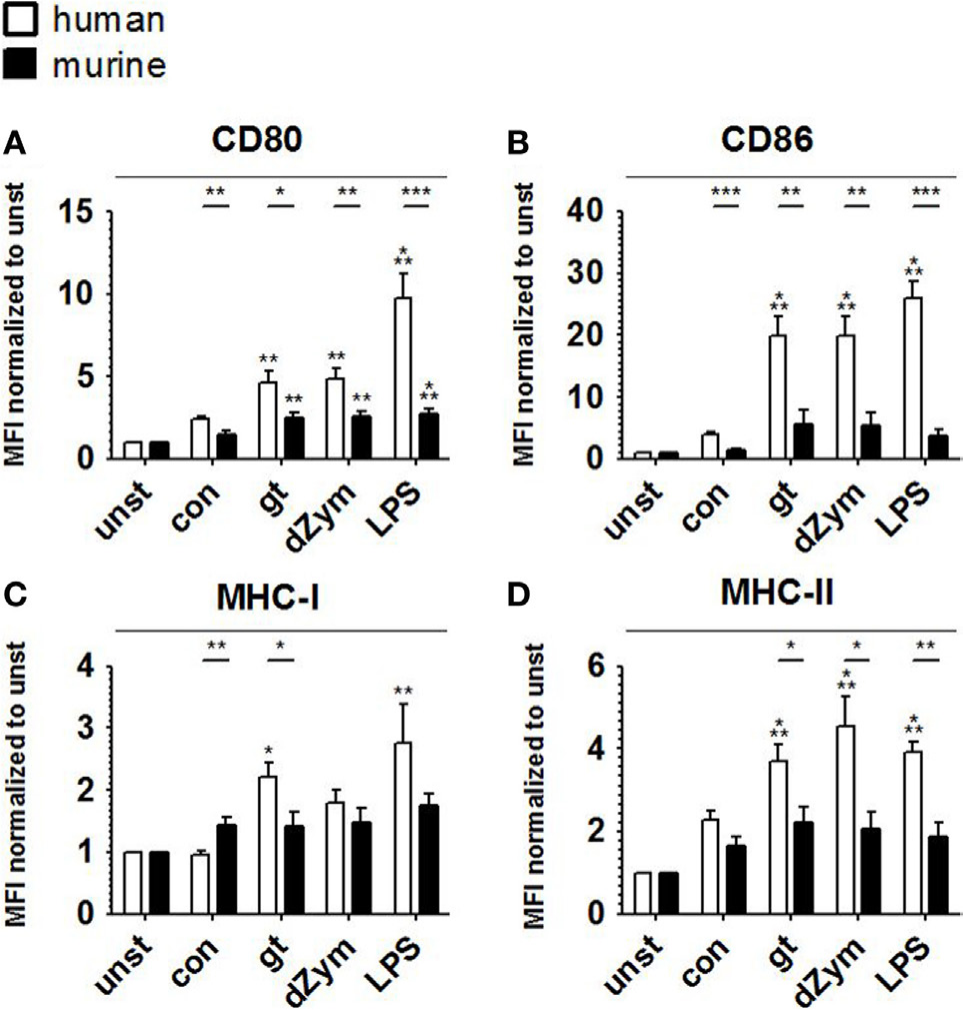
Stronger upregulation of maturation markers and major histocompatibility complex (MHC) molecules upon *Aspergillus fumigatus* stimulation is observed in human moDCs. Surface expression of CD80 **(A)**, CD86 **(B)**, MHC-I **(C)**, and MHC-II **(D)** was analyzed by flow cytometry in human moDCs and murine bone marrow derived dendritic cells after 24 h stimulation with *A. fumigatus* conidia (con) and germ tubes (gt) as well as depleted zymosan (dZym) and LPS. Six human and murine dendritic cell samples were assessed. Mean fold changes of MFI values compared to unstimulated cells (unst) and SDs are shown. Significance between human and mouse specimen was calculated with the unpaired *t*-Test, the comparison to unstimulated specimens is shown as stars above bars and was calculated with ANOVA and Tukey post-test.

To further characterize the response patterns of human and murine DCs confronted with resting and germinated stages of *A. fumigatus*, we analyzed the expression of maturation markers (CD80 and CD86) and MHC molecules (MHC-I and MHC-II) on the cell surface. 24 h of stimulation with inactivated *A. fumigatus* germ tubes, dZym, and LPS led to 1.3-fold upregulation of CD80, CD86, MHC-I, and MHC-II on both human and murine DCs (Figure 6). Upon coculture with all studied stimuli, significantly greater levels of CD80 and CD86 were observed on human moDCs (Figures 6A,B). Coculture with *A. fumigatus* germ tubes led to a significantly higher MHC-I and MHC-II surface expression on human moDCs, whereas conidial stimulation resulted in stronger upregulation of MHC-I in murine BMDCs (Figures 6C,D).

**Figure 7:**
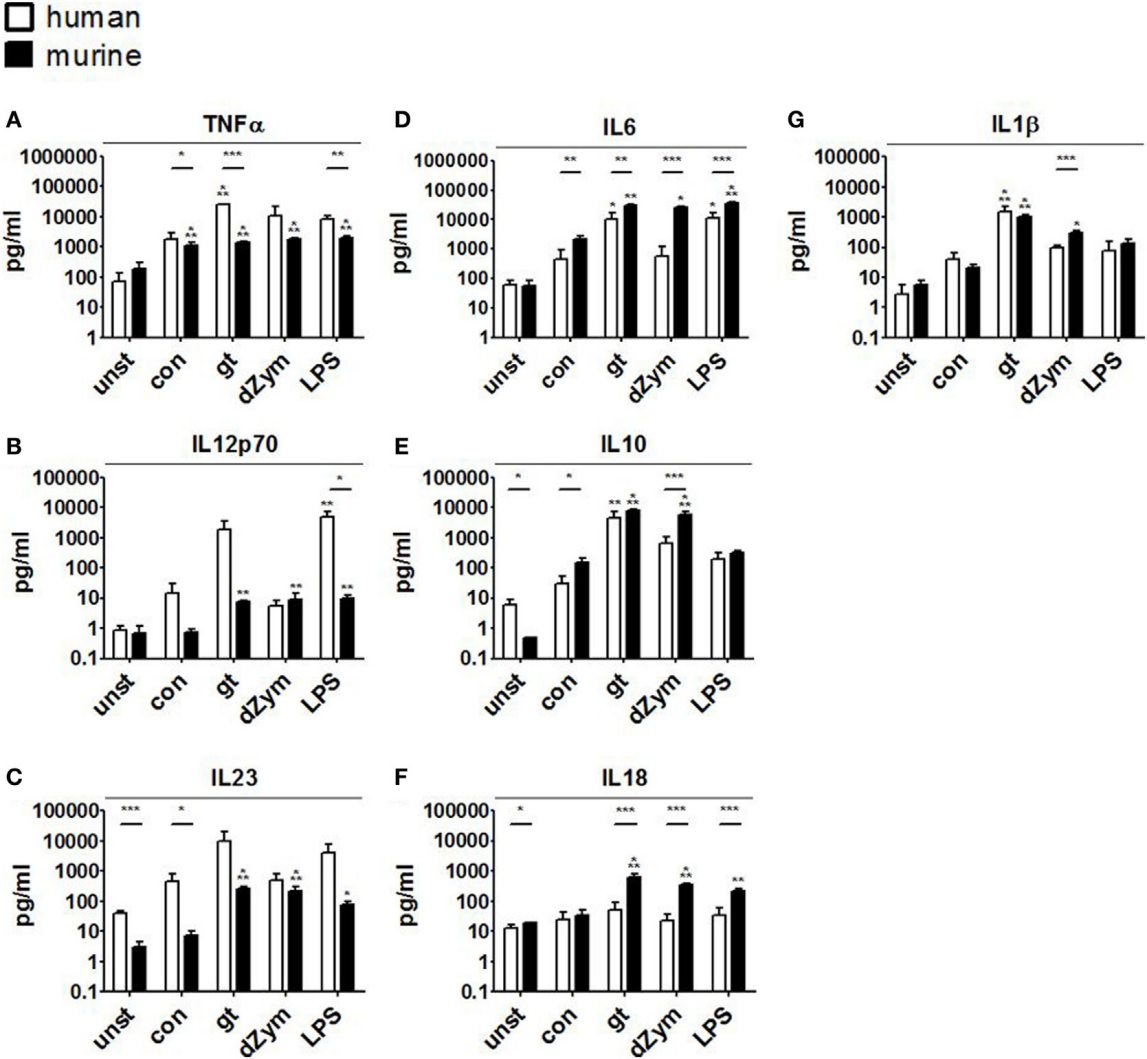
Distinct cytokine response patterns to *Aspergillus fumigatus* are observed in human and murine dendritic cells (DCs). Cytokine concentrations (picograms/milliliter) in culture supernatants of human moDCs and murine bone marrow-derived dendritic cells stimulated with ethanol-inactivated *A. fumigatus* conidia or germ tubes, depleted zymosan (dZym) or LPS were quantified by 13-plex cytokine assays. Four human (white bars) and four murine (black bars) DC samples were assessed. Mean concentrations of TNFα **(A)**, IL12p70 **(B)**, IL23 **(C)**, IL6 **(D)**, IL10 **(E)**, IL18 **(F)**, and IL1β **(G)** are shown. Error bars indicate standard deviations. Significance was calculated as difference to unstimulated specimens with ANOVA and Tukey post-test (stars above of bars) as well as between human and mouse specimens with unpaired *t*-test.

Further, cytokine profiles were studied by bead-based multiplex cytokine assays after 24 h coculture of DCs with *A. fumigatus* conidia, germ tubes, dZym, or LPS. Stimulation with *A. fumigatus* germ tubes or LPS led to release of a broad range of pro-inflammatory cytokines by both human moDCs and murine BMDCs. Human moDCs showed significantly stronger TNFα release in response to these stimuli and a trend toward stronger IL12p70 and IL23 induction. By contrast, murine BMDCs released significantly greater amounts of IL6 and IL18 (Figure 7).

Conidial stimulation resulted in the release of fewer cytokines and lower concentrations than other stimuli. Murine BMDCs showed strong and significant induction of the pro-inflammatory cytokines IL1β, IL6, TNFα, but also increased IL10 release after coculture with conidia. Stimulation of murine BMDCs with dZym led to significantly greater release of IL6, IL10, and IL18, whereas a tendency toward greater TNFα release by human moDCs was observed.

Taken together, these results highlight different response patterns of human moDCs and murine BMDCs confronted with *A. fumigatus*, with distinct cytokine profiles and stronger expression of maturation markers and MHC molecules on human moDCs.

### Fungicidal Activity of PMNs

Colorimetric analysis of the fungal metabolism revealed that 2 h of *A. fumigatus* germ tube–PMN co-cultivation led to markedly increased fungal metabolism while fungal metabolic activity was decreased again after 4 h, compared to unstimulated control samples. No major difference was observed between human and murine PMNs (Figure S2 in Supplementary Material).

## Discussion

*Aspergillus fumigatus* is the most important cause of invasive fungal infections occurring almost exclusively in immunocompromised patients. A key to understanding *A. fumigatus* pathogenicity is knowledge of the interplay between the fungus and the immune system, in particular with the initial innate immune defense consisting of neutrophils, macrophages, and DCs.

In recent decades, several hundred studies characterized the functions of innate immune cells directed against *A. fumigatus* thereby improving the management and treatment of aspergillosis (15). This intensive work was based on *in vitro* studies involving culturing of primary human immune cells, and cell lines and a large variety of animal models including mice, which are frequently used to model human disease and to mimic scenarios of immunocompromised patients.

This study, the first to our knowledge, provides a comparative functional assessment of murine innate immune cell subsets routinely employed in *A. fumigatus in vitro* interaction studies with their human counterparts. Humans and mice differ greatly in their size, lifespan, living conditions, and ecological niches. Even the blood composition between different mouse strains varies widely, while C57BL/6 mice contain 10–25% PMNs and 75–90% lymphocytes (16), CD-1 mice exhibit 15–20% PMNs (300–2,000 cells/μl) and 50–70% lymphocytes (1,000–7,000 cells/μl) (17). In contrast, human blood contains 50–70% PMNs (3,500–7,000 cells/μl) and 20–40% lymphocytes (1,400–4,000 cells/μl) (17). In consequence, a direct translation of murine *in vivo* experimental data to human pathological events often fails due to insufficient similarities in the organization of the immune system of both species (18).

In our study, we isolated and cultivated PMNs, macrophages, and DCs from both mammals and challenged them *in vitro* with *A. fumigatus* using standardized and reproducible working conditions, laboratory protocols and readout assays. We are aware that this *in vitro* comparison is restricted due to the isolated use of single immune cell types while well-established murine models allow a complex view of the pathogenesis of IA. However, the chosen comparative *in vitro* study design nicely illustrates the substantial parallel organization of the human and murine immune response against *A. fumigatus* but also provides examples for functional heterogeneity in the defense against the fungus.

Polymorphonuclear cells are among the first line response against *A. fumigatus*. When attracted by chemokines they leave the blood stream and migrate to the site of infection using a large number of PRRs to recognize and respond to the fungus. This includes the release of soluble antimicrobials, reactive metabolites, cytokines, and phagocytosis of conidia (19). In our experiments, murine and human neutrophils were directly isolated from anticoagulated blood using similar protocols (polysaccharide gradient for human blood and the commercial neutrophil enrichment kit for mice). Murine PMNs exhibited a significantly stronger ROS response to *A. fumigatus* and dZym, a pure dectin-1 agonist, than human PMNs (Figure 1). The role of ROS in the immune defense against *A. fumigatus* is rather unclear. While text-book knowledge indicates that ROS is used by PMNs to kill *A. fumigatus*, more recent reports state that they play a regulatory role as signaling molecules and in the activation of antimicrobial enzymes in the phagolysosome (20). Furthermore, it could be demonstrated only recently that murine lung PMNs trigger programmed cell death with apoptosis-like features in *A. fumigatus* conidia (21).

Furthermore, human PMNs showed strongly elevated IL8 release when stimulated with *A. fumigatus* germ tubes and dZym while murine PMNs secreted a much larger variety of cytokines and chemokines, including Groα, IL6, MIP1α, MIP1β, and TNFα, as well as the anti-inflammatory cytokine IL10. While IL8 is the most important chemoattractant for human neutrophil recruitment, an orthologous counterpart is absent in mice (22). In contrast, mice express CXCL15 as an attractant and other functional homologs of IL8, such as Groα (23).

Interestingly, in a preliminary experiment analyzing PMNs from four healthy human donors and two mice by an XTT metabolic assay, we revealed no major differences quantifying fungal metabolism after 2 and 4 h, respectively. We hypothesize that the increase of fungal metabolism after 2 h of co-cultivation with human and murine PMNs might reflect temporarily higher fungal metabolic activity due to early defense mechanisms of *A. fumigatus*.

Human moDCs were generated from CD14^+^ monocytes using rGM-CSF and rIL4 while murine DCs were derived from bone marrow cultured in R10-medium supplemented with rmGM-CSF. While murine Ly6C^high^ monocytes fail to proliferate (24), proliferating cells mostly represent macrophage-DC progenitors and common monocyte progenitor (25). This observation was described for human CD14^+^ monocytes undergoing moDC differentiation as well (26). Thereby, GM-CSF has major effects on myelomonocytic cells leading to a massive expansion of macrophage–DC-restricted precursors, but very low effects on common dendritic cell precursors (24). In both, humans and mice, Ly6C^high^ monocytes and moDCs are recruited into tissue under inflammatory and infectious conditions thereby initiating T cell priming in the draining lymph nodes, e.g., in the synovia of rheumatoid arthritis patients (27, 28). Thus, murine BMDCs and human moDCs are highly similar and thus can be considered as functional homologs (29, 30).

Both, human and murine DCs were able to phagocytose *A. fumigatus* conidia and germ tubes. Efficient phagocytosis is a prerequisite for adequate antigen processing and presentation to lymphocytes (31). This characteristic property of DCs in the interplay with *A. fumigatus* underlines their relevance in bridging innate and adaptive immunity during mold infections. The more efficient phagocytosis of *A. fumigatus* conidia in human moDCs might be due to the differential size of human and murine DCs or due to mouse-strain specific variabilities in phagocytosic capacity (32).

Human moDCs secreted substantially more of the pro-inflammatory mediators TNFα, MCP1, IL12p70, and IL1β, indicating their profound role in the broad activation of the innate and adaptive immune response. Murine BMDCs released significantly greater amounts of IL6 and IL18. Cenci et al. showed that IL6(−/−) mice were more susceptible to aspergillosis than the wild-type. Susceptibility was associated with increased inflammatory pathology, decreased antifungal effector functions of phagocytes, and impaired development of protective type 1 responses. Exposure to exogenous IL6 restored antifungal effector activity (33). Similarly, simultaneous neutralization of IL18 and IL12 resulted in a significant increase in *A. fumigatus* CFU in murine lung tissue demonstrating its key role in murine defense against *Aspergillus* infection (34).

Secretion of IL10, which suppresses proliferation, cytokine secretion, and costimulatory molecule expression of pro-inflammatory immune cells, differed markedly between human and murine cells. While *A. fumigatus* germ tubes and dZym led to a 100- and 60-fold induction of the IL10 secretion of human macrophages compared to unstimulated control cells, stimulation of murine macrophages led to lower increases (with germ tubes 14-fold, with dZym 14-fold). Interestingly, basal secretion of IL10 from unstimulated macrophages into the culture medium was over 20-fold higher in human cells than in their murine counterparts. Human and mouse IL10 have roughly 73% sequence homology and are secreted as 178-amino acid proteins (35). It is unknown whether both cytokines have identical binding capacity to their receptors, stability, and degradation behavior.

Dectin-1, a β-glucan receptor, is expressed on monocytes, macrophages, DCs, neutrophils, and eosinophils (36). Brown et al. demonstrated that dZym, which was treated with hot alkali to remove its TLR-stimulating properties, triggers expression of pro-inflammatory cytokines *via* Dectin-1 signaling (37). In mice, sensing of *C. albicans* by Dectin-1 results in ingestion and killing of the fungus and the induction of an early inflammatory response, which results in the recruitment and activation of other immune cells to the infection site. Dectin-1 deficiency leads to increased susceptibility to systemic and mucosal candidiasis (38). In contrast, human Dectin-1 deficiency causes diminished cytokine responses, while alternative receptors are then responsible for fungal uptake and subsequent killing. Our results demonstrate inverse Dectin-1 surface expression after exposure to *A. fumigatus*. After 1 h of coculture, surface expression of Dectin-1 decreased on human moDCs, with a more pronounced effect after stimulation with germ tubes (Figure 5A). By contrast, increased Dectin-1 expression was observed on murine BMDCs stimulated with *A. fumigatus* germ tubes (Figure 5B). We hypothesize that downregulation of Dectin-1 exposure on human moDCs prevents severe and uncontrolled inflammatory reactions while on murine BMDCs, Dectin-1 expression is required for continuous ingestion and killing of the fungus. This is consistent with our previous data showing that anti-Dectin-1 antibody treatment of human moDCs as well as *ex vivo* myeloid DCs subsequently inhibited secretion of pro-inflammatory cytokines after contact with *A. fumigatus, C. albicans*, or zymosan (4, 39). This is also consistent with the observation that naïve mice lacking Dectin-1 show reduced killing of conidia and a higher mortality rate than wild-type mice (40).

Taken together, after 65 million years of independent evolution (5), immune systems of both species differ substantially. Mice are perfectly adapted to their relatively short lifespan of 2–3 years and their natural habitat on the ground. In the defense against invading fungi, resistance mechanisms and an efficient control over pathogens dominate in humans, while tolerance determines the immune response in mice (41). Our comparative functional assessment of selected human and murine innate immune cell subsets using standardized and reproducible working conditions, laboratory protocols, and readout assays underlined this hypothesis. We were able to provide examples for functional heterogeneity of mammalian innate immune cell populations in their *in vitro* response against the pathogenic mold *A. fumigatus*. These specific differences should be carefully considered in future comparative discovery and validation studies. Furthermore, they highlight potential limitations in the direct transferability of murine host–pathogen interaction studies. Additional studies are highly warranted comparatively employing murine and human cell populations to further study mold immunopathology and to evaluate new diagnostic and therapeutic strategies. Moreover, work on humanized mouse models or the development of synthetic human models might help to avoid difficulties in translating and harmonizing data across different mammalian species.

## Ethics Statement

Use of whole blood specimens from healthy volunteers was approved by the University Hospital of Wuerzburg Ethical Committee (#302/15). Written informed consent was obtained and data analysis was performed anonymously. Mouse husbandry and experimental procedures were conducted in accordance with institutional guidelines and with the approval of the Committee on Animal Research of the regional government (Regierung von Unterfranken, Wuerzburg, Germany).

## Author Contributions

A-MH and JaL designed the study, performed experiments, performed data analyzes, and wrote the manuscript. AS performed experiments. SW and ML analyzed the data and contributed to the manuscript. CM, ME, and KC provided discussions, technical assistance and contributed to the manuscript. HE and JuL developed concepts, supervised the study, and wrote the manuscript.

## Conflict of Interest Statement

The authors declare that the research was conducted in the absence of any commercial or financial relationships that could be construed as a potential conflict of interest.

## Supplementary Material

Figure S1 Comparison of cytokine response patterns to *Aspergillus fumigatus* germ tubes. Cytokine secretion into the culture medium was analyzed by multiplex bead-based assay upon stimulation of murine or human dendritic cells (DCs), neutrophils [polymorphonuclear cells (PMNs)], or macrophages with *A. fumigatus* germ tubes. Fold changes compared to unstimulated samples are indicated by gray scale. OOR (out of range) indicates that >50% of samples did not meet the limits of detection and thus fold changes could not be calculated reliably.

Figure S2 Impact of human and murine polymorphonuclear cells (PMNs) on fungal XTT metabolism. PMNs were isolated from whole blood of four healthy human donors and two mice as described in the Section “Materials and Methods.” After 2 and 4 h of coculture at 37°C, hypotonic lysis of PMNs was performed. Supernatants were carefully removed and 200 µl HBSS supplemented with 400 µg/ml of 2,3-bis-(2-methoxy-4-nitro-5-sulphenyl)-(2 H)-tetrazolium-5-carboxanilide (XTT) and 50 µg/ml of coenzyme were added. After 90 min incubation at 37°C and centrifugation at 300*g* for 5 min, 100 µl supernatant of each well were transferred to a 96-well plate and OD_450_ was measured in a microplate reader. Fold changes of fungal XTT metabolism due to the presence of PMNs was calculated according to the following formula: $\text{Fold change}=({\text{OD}}_{{\;}_{450}}^{{\;}^{\text{PMN}+\text{Fungus}}}-{\text{OD}}_{{\;}_{450}}^{{\;}^{\text{PMN}}})/({\text{OD}}_{{\;}_{450}}^{{\;}^{\text{Fungus}}}-{\text{OD}}_{{\;}_{450}}^{{\;}^{\text{Blank}}}).$ Individual results for each human and murine PMN sample and mean values (black horizontal bars) are shown in the figure.
