# Supplementary figures and images for: Human and Murine Innate Immune Cell Populations Display Common and Distinct Response Patterns during Their In Vitro Interaction with the Pathogenic Mold Aspergillus fumigatus
Source: Front Immunol. 2017 Dec 6;8:1716. doi: 10.3389/fimmu.2017.01716 (PMC5723658; doi:10.3389/fimmu.2017.01716)

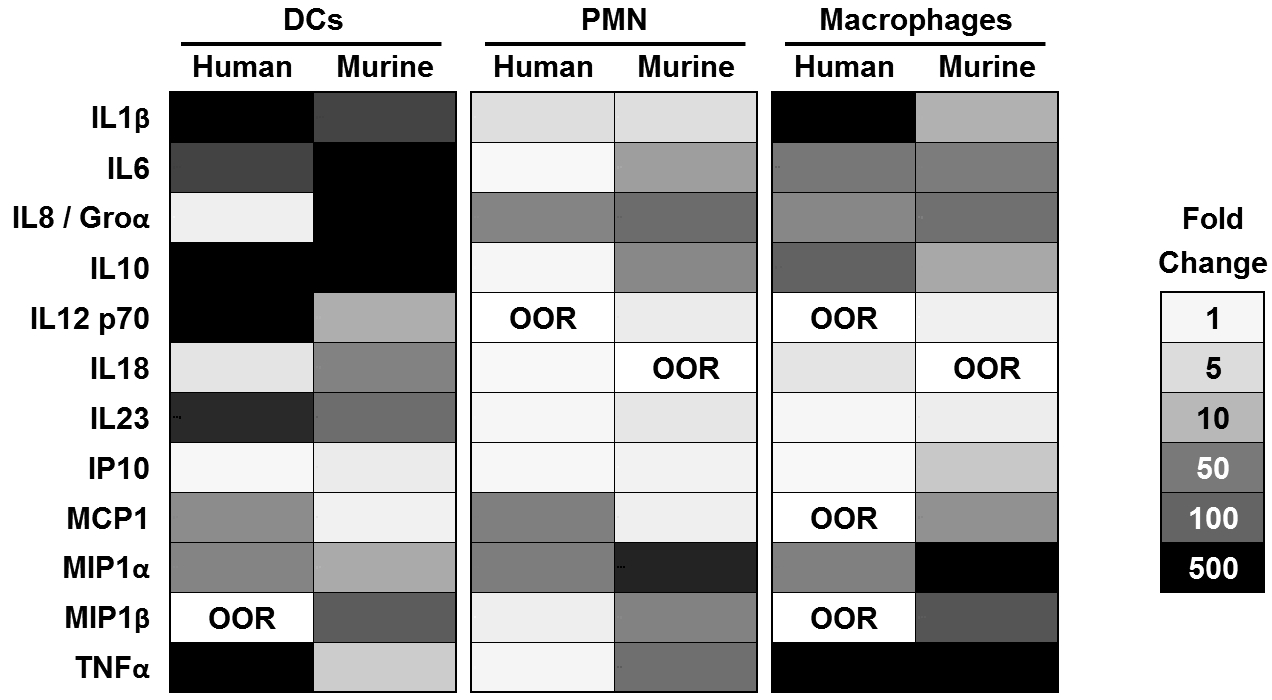

Supplement: Figure S1 — Comparison of cytokine response patterns to Aspergillus fumigatus germ tubes. Cytokine secretion into the culture medium was analyzed by multiplex bead-based assay upon stimulation of murine or human dendritic cells (DCs), neutrophils [polymorphonuclear cells (PMNs)], or macrophages with A. fumigatus germ tubes. Fold changes compared to unstimulated samples are indicated by gray scale. OOR (out of range) indicates that >50% of samples did not meet the limits of detection and thus fold changes could not be calculated reliably. [file image_1.jpeg]

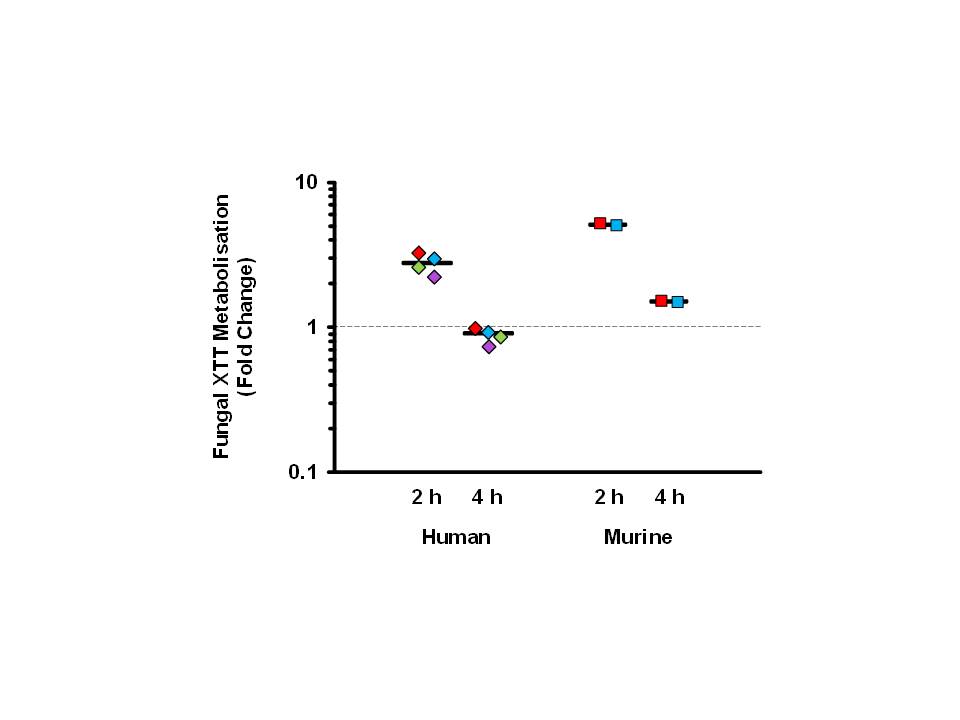

Supplement: Figure S2 — Impact of human and murine polymorphonuclear cells (PMNs) on fungal XTT metabolism. PMNs were isolated from whole blood of four healthy human donors and two mice as described in the Section “Materials and Methods.” After 2 and 4 h of coculture at 37°C, hypotonic lysis of PMNs was performed. Supernatants were carefully removed and 200 µl HBSS supplemented with 400 µg/ml of 2,3-bis-(2-methoxy-4-nitro-5-sulphenyl)-(2 H)-tetrazolium-5-carboxanilide (XTT) and 50 µg/ml of coenzyme were added. After 90 min incubation at 37°C and centrifugation at 300g for 5 min, 100 µl supernatant of each well were transferred to a 96-well plate and OD450 was measured in a microplate reader. Fold changes of fungal XTT metabolism due to the presence of PMNs was calculated according to the following formula: Fold change=(OD 450 PMN+Fungus−OD 450 PMN)/(OD 450 Fungus−OD 450 Blank). Individual results for each human and murine PMN sample and mean values (black horizontal bars) are shown in the figure. [file image_2.jpeg]
